# Insecticide resistance status of malaria vectors in Lao PDR

**DOI:** 10.1371/journal.pone.0175984

**Published:** 2017-04-24

**Authors:** Sébastien Marcombe, Julie Bobichon, Boutsady Somphong, Nothasin Phommavan, Santi Maithaviphet, Simone Nambanya, Vincent Corbel, Paul T. Brey

**Affiliations:** 1 Institut Pasteur du Laos, Ministry of Health, Vientiane, Lao PDR; 2 Center for Malariology, Parasitology and Entomology, Ministry of Health, Vientiane, Lao PDR; 3 Institut de Recherche pour le Développement (IRD), Maladies Infectieuses et Vecteurs, Ecologie, Génétique, Evolution et Contrôle (MIVEGEC, UM1-CNRS 5290-IRD 224), Montpellier, France; New Mexico State University, UNITED STATES

## Abstract

Knowledge on insecticide resistance in *Anopheles* species is a basic requirement to guide malaria vector control programs. In Lao PDR, vector control relies on insecticide residual spraying (IRS) and impregnated bed-nets (ITNs) with the use of pyrethroids. Here, the susceptibility of *Anopheles* species, including several malaria vectors (*An*. *maculatus* and *An*. *minimus*), to various insecticides was investigated in ten provinces of Lao PDR through a north-south transect. Bioassays were performed on field caught female mosquitoes using the standard WHO susceptibility tests with DDT (4%), deltamethrin (0.05%) and permethrin (0.75%). In addition, the DIIS6 region of the para-type sodium channel gene was amplified and sequenced to identify knockdown resistance mutations (*kdr*). Resistance to DDT and permethrin was detected in suspected malaria vectors, such as *An*. *nivipes* and *An*. *philippinensis* in Lao PDR. Resistance to the formerly used DDT was found in a population of *An*. *maculatus* s.l. from Luang Prabang province. No resistance to pyrethroids was found in primary vectors, indicating that these insecticides are still adequate for malaria vector control. However, high resistance levels to pyrethroids was found in-vector species and reduced susceptibility to permethrin in *An*. *minimus* and *An*. *maculatus* was reported in specific localities which raises concerns for pyrethroid-based control in the future. No *kdr* mutation was found in any of the resistant populations tested hence suggesting a probable role detoxification enzymes in resistance. This study highlights the necessity to continue the monitoring of insecticide susceptibility to early detect potential occurrence and/or migration of insecticide resistance in malaria vectors in Lao PDR.

## Introduction

Vector borne diseases account for approximately 17% of the estimated global burden of infectious diseases and are the major causes of illness and death in tropical and sub-tropical countries [1]. The most deadly vector-borne disease, malaria, caused an estimated 429,000 deaths in 2016, mostly in the WHO African Region (90%), followed by the WHO South-East Asia Region (7%). The malaria burden in the Greater Mekong Sub-region (GMS) remains a major public health problem impacting on the health and lives of a large proportion of people [1]. In the Lao People’s Democratic Republic (Lao PDR) malaria is endemic, but intensity of transmission is heterogeneous, with more intense transmission in remote and forested areas particularly in the south [2]. Although Lao PDR has reduced malaria incidence by 50% since 2000, recrudescence of cases has been reported since 2011, with more than 260,000 cases reported in 2015 [1]. In Lao PDR, as in most GMS countries, malaria vector control relies on the use of insecticide treated materials (i.e. long-lasting insecticide treated nets [LLIN]) and indoor residual spraying (IRS) [1]. Between 1999 and 2000, 40,000 ITNs (50% LLINs) were distributed in 4 Lao provinces [3] and ten years later more than 90% of the country was covered with LLINs [4]. Before the use of ITNs, residual spraying with DDT (organochlorine family) was the method of choice for malaria control. Use of DDT was stopped in 1990 (officially banned in 2010, [5]) and since then insecticides from the pyrethroid family (eg permethrin, deltamethrin, alpha-cypermethrin and lambda-cyhalothrin) are utilized for IRS and/or ITNs [4].

As in many countries, wide implementation of residual insecticides have contributed to significantly reducing the burden of malaria in Lao PDR [3,6]. However, the emergence of insecticide resistance (IR) in many malaria-affected countries poses a significant challenge to the continued success of these vector control methods, [1,7]. In 2008, during the MALVECASIA project, Van Bortel and colleagues reported insecticide resistance in major malaria vectors (i.e. *Anopheles dirus*, *An*. *minimus* and *An*. *epiroticus*) in the neighboring countries of Lao PDR such as Cambodia, Vietnam and Thailand [8]. At that time, no resistance was found in malaria vectors in Lao PDR, but resistance to DDT was suspected in the non-vector species, *An*. *vagus*.

Insecticide resistance in insects is caused by a reduced penetration of the insecticide due to a modification of the cuticle [9], increased activity or level of detoxification enzymes (metabolic-based resistance) and a reduction of the sensitivity of the target site (target-site resistance). The main target site resistance mechanism in *Anopheles* mosquitoes involve three non-synonymous mutations (L1014F, L1014C, and L1014S) at the kdr codon L1014 of para-type sodium channel gene that cause a resistance to pyrethroid insecticides [10–12]. In Asia these mutations have been reported in several *Anopheles* species from India [13], Sri Lanka [14], Indonesia [15], China [12], Vietnam and Cambodia [16]. Metabolic-based resistance involves three major detoxification enzyme families and is now considered a key resistance mechanism in mosquitoes [7]. Up-regulation of esterases and cytochrome P450 was observed in pyrethroid resistant *Anopheles* populations from Thailand and Vietnam [17,18] hence suggesting the involvement of metabolic resistance in pyrethroid resistance.

The present study was conducted in the framework of a nationwide entomology surveillance (MALVEC project) aiming at filling knowledge gaps in malaria vector bionomic and insecticide resistance in Lao PDR. Here we report the insecticide resistance status of *Anopheles* populations to insecticides historically (DDT) or currently used for malaria vector control (i.e. permethrin, deltamethrin) in 10 provinces of Lao PDR over 2 years’ time.

## Material and methods

### Mosquito collection and identification

**Fig 1:**
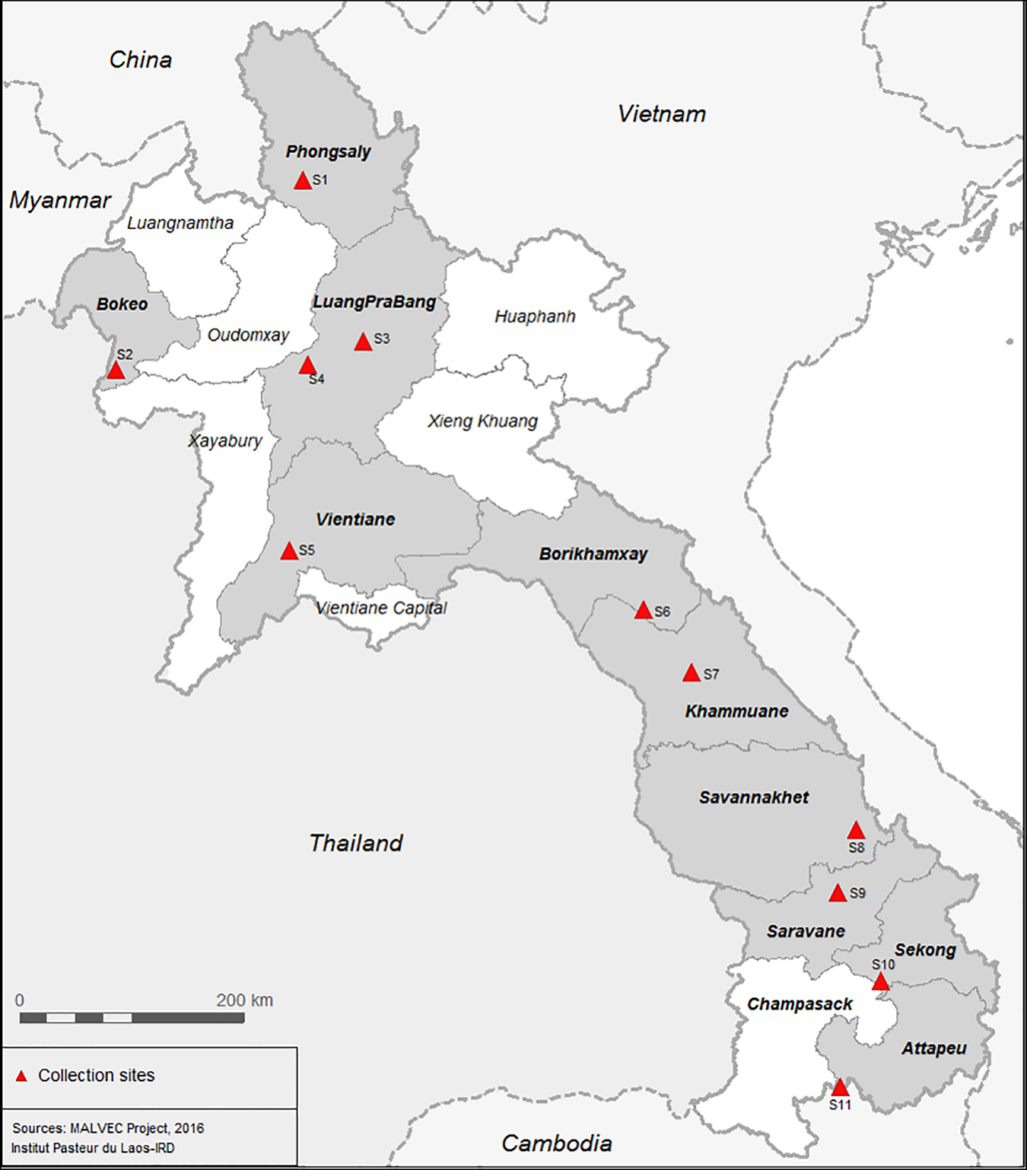
Location of mosquito collection sites during 2013–2015 in Lao PDR.

Ten villages from ten provinces in Lao PDR were selected for the study (Table 1 and Fig 1). Four mosquito surveys were carried out during the rainy (June to October) and dry (January to May) seasons of 2014 and 2015. Mosquito collections were done at the same sites for the duration of the study period except in Luang Prabang province where the study site was changed from Sopjak village (S3—Table 1 and Fig 1) to Na village (S4—Table 1) due to accessibility issues. Mosquitoes were collected by human landing catches (HLC) and cow bait collections (CBC) from 18:00 to 06:00 during four consecutive nights. Mosquitoes were stored in cups and were provided with sugar solution until morphological identification. The next morning following collections, mosquitoes from both collection methods were pooled and morphologically identified to species or species group/complex in field laboratory, using microscopes and appropriate identification keys [19]. After identification, mosquitoes were separated by species, kept in separate cages with humid conditions until sufficient numbers were obtained, when possible, for insecticide susceptibility bioassays. The waiting period to obtain a sufficient number of mosquitoes for the tests was maximum two nights to avoid the side effects of being kept in cages for too long.

**Table 1:** Mosquito collection sites in Lao PDR.

Site number	Province	District	Village	Latitude	Longitude
S1	Phongsaly	Bountai	Boulykao	21.33778	102.08247
S2	Bokeo	Paktha	Hadsa	19.92268	100.58148
S3	Luang Prabang	Pakseng	Sopjak	20.13477	102.55834
S4	Luang Prabang	Chomphet	Na	19.96715	102.11792
S5	Vientiane Pro.	Feuang	Na-ang	18.55996	101.97389
S6	Borlikhamxay	Khamkeut	Phameung	18.11425	104.80229
S7	Khammouane	Gnommalath	Koutphadang	17.63663	105.17795
S8	Savannakhet	Nong	Sadi	16.43901	106.50284
S9	Saravane	Toomlarn	Katao	15.95187	106.35285
S10	Sekong	Lamam	Lavynoy	15.27291	106.69748
S11	Attapeu	Sanamxay	Hadoudomxay	14.45668	106.36727

### Susceptibility bioassay

Insecticide susceptibility bioassays (tube tests) were performed on collected mosquitoes following WHO protocols to assess potential insecticide resistance [20]. Adult females were exposed to WHO discriminating dosages of deltamethrin (0.05%), permethrin (0.75%), and DDT (4%). The insecticide-impregnated papers were supplied by the Vector Control Research Unit, Universiti Sains Malaysia and were not used more than five times. Mosquitoes exposed to untreated papers were used as control. Mosquitoes were exposed for 60 minutes to estimate the knockdown time (KDT_50_) using log-time and probit mortality regression [21]. For each tests, knock-down mosquito numbers were estimated every 5 minutes. Mortality was recorded 24 hours after exposure. Mortality of the exposed mosquitoes was calculated by summing the number of dead mosquitoes across all replicates (usually 25 mosquitoes in 4 tubes; in total, 100 mosquitoes when possible) and expressing this as a percentage of the total number of exposed mosquitoes:

$\text{Observed}\;\text{mortality}=\left(\frac{Total\;number\;of\;dead\;mosquitoes}{Total\;tested}\right)\times 100$ When the control mortality was higher than 20%, the test was discarded. When control mortality was greater than 5% but less than 20%, the observed mortality was corrected using Abbott’s formula [22]. A weighted mean was applied to summarize the mortality between the different seasons (rainy and dry seasons 2014–2015). According to WHO criteria, a mosquito population was considered resistant whether the 24-hour mortality was <90%. Resistance was suspected when mortality was between 90% and 98% and the population was susceptible when the mortality was >98%. Mosquitoes from the bioassays were stored either in RNAlater® when alive or in silica gel when dead for subsequent laboratory analysis.

### Detection of *kdr* mutations

Mosquitoes were screened for *kdr* mutations in the DIIS6 region of the para-type sodium channel gene VGSC and the detection was done by PCR methods as described by Syafruddin et al. [15]. DNA from resistant (survivors to insecticide exposure) and control mosquitoes was extracted, amplified and then sequenced for identification of the known resistant mutations present in *Anopheles spp*. (e.g. L1014F/L1014S). DNA was extracted from each mosquito screened for *kdr* mutation using a commercial extraction kit (NucleoSpin® 96 Virus, Machery Nagel GmbH & Co. KG, Germany) according to manufacturer instructions. Extracted DNA was send to a commercial company (Macrogen, Korea) for purification and sequencing using the following primers: Ag-F_*kdr* (5’ GAC CAT GAT CTG CCA AGA TGG AAT 3’) and An.*kdr*_R2 (5’ GAG GAT GAA CCG AAA TTG GAC 3’). Sequencing analysis was performed using BioEdit software program version 7.2.5.

### Sibling species identification

The same DNA samples used for the *kdr* screening were used for the sibling species molecular identification of the resistant mosquitoes. Specific primers were designed to distinguish between sibling species among the Dirus, Maculatus and Minimus group/complexes (Marcombe et al. in prep). All selected mosquitoes that belonged to this group/complexes were identified by using a single multiplex PCR method proposed by Walton [23,24], and Garros et al. [25]. To check whether the PCR generated the anticipated DNA fragment, agarose electrophoresis gel was used for size separation of PCR product with comparison of the DNA ladder.

### Ethical considerations

Ethical clearance for mosquito collection was obtained from Lao PDR Council of Medical Science National Ethics Committee (authorization No033/NECHR, 05/07/2013). Each collector signed an informed consent form and received a Japanese Encephalitis vaccination (IMOJEV®MD, GPO-MBP Co., Ltd).

## Results

### Susceptibility bioassays

More than 14,000 adult mosquitoes representing 25 different *Anopheles* species were collected during the study. Among them 78% were collected on animal. A total of 142 susceptibility tests were performed during the study. Table 2 shows the number of mosquitoes tested for each species and insecticide. A total of 3,997 mosquitoes were phenotyped including 1,449, 1,136 and 1,392 specimen for permethrin, deltamethrin and DDT, respectively. Primary and secondary vectors were tested as well as non-vectors representing a total of 10 *Anopheles* species. A total of 585 *An*. *maculatus* and 746 *An*. *minimus* (primary vectors) were bio-assayed. In overall, *Anopheles tessellatus* was the less representative species (with only 23 mosquitoes tested) whereas *An*. *vagus* was the most representative one (961 specimens tested).

**Table 2:** Number of *Anopheles* tested of each species against three insecticides using WHO susceptibility tests, Lao PDR, 2014–2015.

Species	Permethrin (0.75%)	Deltamethrin (0.05%)	DDT (4%)	Total Tested
*An*. aconitus**	162	105	78	345
*An*. *hyrcanus s*.*l*.	45	38	40	123
*An*. *kochi*	38	12	59	109
*An*. *maculatus s*.*l*.*	193	162	230	585
*An*. *minimus s*.*l*.*	290	244	212	746
*An*. *nivipes s*.*l*.**	233	273	267	773
*An*. philippinensis**	126	NA	104	230
*An*. *tessellatus*	9	NA	14	23
*An*. *umbrosus*	39	27	16	82
*An*. *vagus*	314	275	372	961
**Total Tested**	**1,449**	**1,136**	**1,392**	**3,977**

*Primary vector.

**Secondary vector.

Note: A primary vector is a species of *Anopheles* mainly responsible for transmitting malaria in any particular circumstance. A secondary vector is thought to play a lesser role in transmission than the principal vector; capable of maintaining malaria transmission at a reduced level or at particular period of the year [26].

It was not always possible to bioassay the recommended numbers of mosquitoes (i.e 100 specimens per species) due to the low density of mosquitoes collected in some surveys (i.e. weather conditions and mortality during collections). Nevertheless we decided to include all bioassays results (n<100) considering the paucity of insecticide resistance data in Lao PDR.

Table 3 shows the mortality of *Anopheles* species after 24h post exposure to permethrin, deltamethrin and DDT from the 10 selected provinces of Lao PDR. The number of female mosquitoes tested per species, per province and per insecticide varied from 9 (*An*. *tessellatus*) to 290 (*An*. *minimus*) specimens. No resistance to pyrethroids was detected in the primary vectors, i.e. Dirus s.l., Minimus s.l. and Maculatus s.l. However, suspected resistance to permethrin was reported in *An*. *maculatus s*.*l*. from Phongsaly and Luang Prabang (site 4, Chomphet district, Table 1) with 98 and 97% mortality, respectively. The test performed in Phongsaly should be confirmed. *Anopheles maculatus s*.*l*. populations from Luang Prabang province showed resistance with 86% mortality. DDT resistance was also suspected in *An*. *maculatus s*.*l*. in Saravane (97%) and Attapeu (92%) provinces. No resistance to pyrethroids was detected in the secondary vectors but *An*. *nivipes* and *An*. *philippinensis* populations showed high resistance levels to DDT in Khammouane provinces (0% and 33% mortality, respectively). Among the non-vectors species tested only *An*. *vagus* showed resistance to all 3 insecticides with mortalities ranging from 34 to 61%. *Anopheles umbrosus* showed both permethrin and DDT resistance at Bokeo province.

**Table 3:** Susceptibility status of *Anopheles* species from the 10 provinces of Lao PDR to pyrethroids and DTT.

Province	*Anopheles* species	Percentage mortality (N, Status)		
		Permethrin (0.75%)	Deltamethrin (0.05%)	DDT (5%)
Bokeo	*An*. minimus*	NA	NA	100 (16, S)
	*An*. *kochi*	100 (10, S)	100 (12, S)	100 (10, S)
	*An*. *umbrosus*	**86 (171, R)**	100 (27, S)	**63 (16, R)**
	*An*. *vagus*	94 (185, RS)	**79 (136, R)**	**61 (166, R)**
Phongsaly	*An*. maculatus*	98 (41, RS)	100 (29, S)	100 (68, S)
	*An*. minimus*	100 (27, S)	NA	100 (10, S)
	*An*. *kochi*	100 (11, S)	NA	**82 (12, R)**
Luang Prabang 1	*An*. *hyrcanus*	100 (16, S)	100 (13, S)	**90 (20, R)**
Luang Prabang 2	*An*. maculatus*	97 (51, RS)	100 (16, S)	**86 (39, R)**
	*An*. minimus*	100 (27, S)	100 (31, S)	98 (53, S)
	*An*. nivipes**	100 (46, S)	100 (43, S)	**89 (42, R)**
	*An*. *vagus*	**89 (47, R)**	90 (21, RS)	**54 (46, R)**
Vientiane Pro.	*An*. minimus*	100 (134, S)	100 (173, S)	100 (97, S)
	*An*. aconitus**	100 (205, S)	100 (187, S)	100 (97, S)
	*An*. *hyrcanus*	100 (29, S)	100 (25, S)	100 (30, S)
	*An*. *nivipes*	100 (42, S)	100 (40, S)	100 (58, S)
	*An*. *tessellatus*	100 (9, S)	NA	**14 (15, R)**
Borlikhamxay	*An*. minimus*	100 (25, S)	NA	NA
	*An*. nivipes**	100 (11, S)	NA	100 (16, S)
	*An*. philippinensis**	100 (26, S)	NA	100 (29, S)
Khammouane	*An*. nivipes**	NA	100 (100, S)	**0 (25, R)**
	*An*. philippinensis**	100 (100, S)	NA	**33 (75, R)**
	*An*. *vagus*	**89 (26, R)**	NA	**61 (22, R)**
Saravane	*An*. maculatus*	100 (37, S)	100 (39, S)	97 (31, RS)
Sekong	*An*. *kochi*	NA	NA	100 (23, S)
	*An*. nivipes**	90 (50, RS)	100 (49, S)	100 (77, S)
	*An*. *vagus*	100 (30, S)	95 (62, RS)	**52 (87, R)**
Attapeu	*An*. maculatus*	100 (80, S)	100 (78, S)	92 (92, RS)
	*An*. minimus*	100 (41, S)	100 (40, S)	100 (19, S)
	*An*. nivipes**	100 (84, S)	100 (66, S)	95 (49, RS)
	*An*. *kochi*	100 (17, S)	NA	**86 (14, R)**
	*An*. *vagus*	95 (58, RS)	**90 (56, R)**	**34 (36, R)**

S, susceptible; RS, resistance suspected; R, resistant; NA, not available.

*Primary vector.

**Secondary vector.

### Knock down time

**Fig 2:**
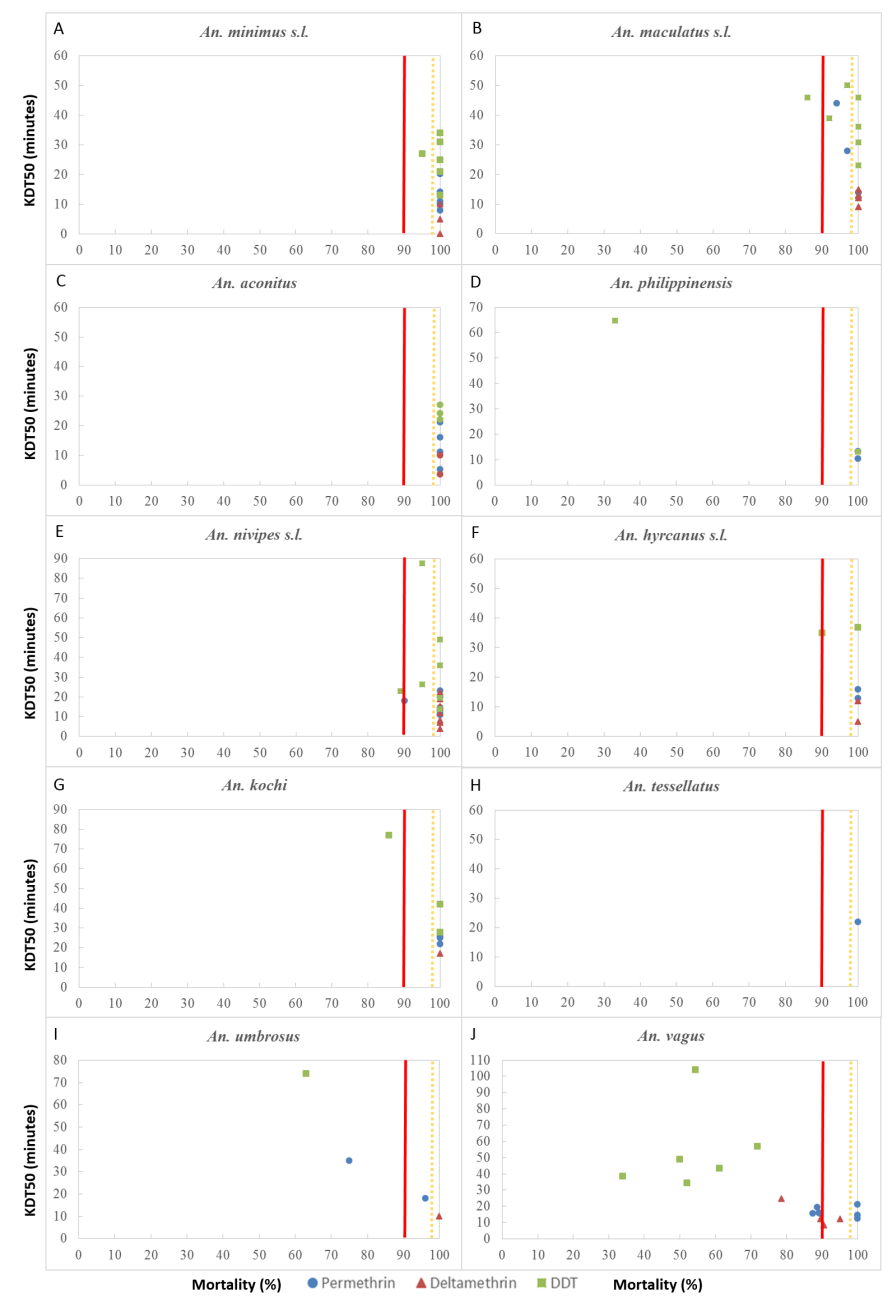
Time for 50% knockdown (KDT_50_) versus mortality for 10 *Anopheles* species from Lao PDR against 3 insecticides (permethrin 0.75% designated with a blue circle, deltamethrin 0.05%designated with a red triangle, DDT 4% designated with a green square). The solid red line indicate the WHO threshold for insecticide resistance (<90% mortality) The orange dotted line indicate suspected resistance (90%<mortality<98%).

The relationship between KDT_50_ and observed mortality in the 10 *Anopheles* species and the three insecticides is shown in Fig 2. The S1 Table shows the detailed data for each test (i.e. KDT_50/95_ and their 95% confidence intervals, the goodness of fit of the tests and the slopes of the regression lines). For all species combined, the exposure time to obtain 50% knockdown ranged from 5 to 44 minutes for permethrin (i.e. *An*. *aconitus* in Vientiane and *An*. *maculatus* in Luang Prabang, respectively), from 4 to 25 minutes for deltamethrin (i.e. *An*. *aconitus* in Vientiane and *An*. *vagus* in Bokeo) and from 13 to 104 min for DDT (i.e. *An*. *minimus* in Phongsaly and *An*. *vagus* in Luang Prabang). Interestingly, *An*. *maculatus* populations from Phongsaly, Luang Prabang and Attapeu provinces showing suspected resistance or resistance to permethrin and DDT had higher KDT_50_ than the susceptible populations collected in the other provinces (Fig 2A). However, in overall, no significant correlations between mortality rates and KDT_50_ was observed for all insecticides and *Anopheles* species.

### Sibling species and *Kdr* mutation

PCR of sibling species were conducted on 4,247 mosquitoes from bioassays representing 13 different *Anopheles* group/species. Regarding malaria vectors only, there was 5.4% of misidentification between the morphological and the molecular methods. The most common misidentifications were between *An*. *minimus s*.*l*. and its related species *An*. *aconitus* due to overlapping morphological characters. Among the Maculatus group, sibling species were *An*. *maculatus s*.*s*, *An*. *rampae*, *An*. *sawadwongporni*, and *An*. *dravidicus* (N = 16, 13, 8 and 6, specimens respectively). Among the Minimus complex, we found *An*. *minimus s*.*s*., *An*. *pampanai* and *An*. *aconitus* (N = 9, 10 and 6 specimens, respectively. No *kdr*-resistant alleles (L1014F/L1014S) were found in any of the 279 specimens screened (Table 4). The crude data can be found at http://malvec.pasteur.la.

**Table 4:** *Kdr* mutation screening in *Anopheles* species after molecular identification.

Species	Allelic Frequency		
	L1014 wild (TTA or CTA)	1014F (TTT)	1014S (TCA)
*An*. *aconitus*	6	0	0
*An*. *dravidicus*	6	0	0
*An*. *kochi*	6	0	0
*An*. *maculatus s*.*s*	16	0	0
*An*. *minimus s*.*s*.	9	0	0
*An*. *nivipes s*.*l*.	26	0	0
*An*. *pampanai*	10	0	0
*An*. *philippinensis*	38	0	0
*An*. *rampae*	13	0	0
*An*. *sawadwongporni*	8	0	0
*An*. *tesselatus*	10	0	0
*An*. *umbrosus*	3	0	0
*An*. *vagus*	128	0	0
**Total**	**279**	**0**	**0**

## Discussion

The purpose of this study was to evaluate the pattern of insecticide resistance in malaria vectors in ten provinces of Lao PDR to guide malaria vector control policies. It is obvious that collecting in only one village per province is not fully representative of the actual resistance pattern in Lao PDR, but these results provide a general trend of the resistance in the malaria vectors.

During three years follow up, no resistance to pyrethroids was detected in malaria vectors in Lao PDR according to WHO guidelines. However, resistance or suspected resistance to DDT was found in malaria vector species, as well as in non-vector species in several provinces. The primary vector *An*. *minimus* was susceptible to all the insecticides except in Luang Prabang province where reduced susceptibility to DDT was noted. *An*. *maculatus* populations were also susceptible in all provinces except in Phongsaly and Luang Prabang where suspected resistance to permethrin was reported (mortality>90%). Previous study conducted in Vientiane province in 2005, at the same location as our study (Na-ang village), showed full susceptibility of *An*. *minimus* to DDT, permethrin and deltamethrin [8]. All together these findings suggest that *An*. *minimus* and *An*. *maculatus* are still under low selection pressure by public health insecticides in Lao PDR. This could be explained by the highly zoophilic and exophagic preferences of these species that may limit their exposure time to residual insecticides [27]. In overall, our results suggest that LLINs or IRS that utilize pyrethroids are still effective at protecting people from indoor *Anopheles* bites and should be deployed widely especially in the southern part of the country where malaria is endemic. However, we recommend the use of new insecticides with different modes of action (e.g. organophophates and carbamates) for vector control in Phongsaly and Luang Prabang provinces where suspected resistance to permethrin was detected in *An*. *maculatus*. Furthermore, higher levels of pyrethroid resistance in the non-vector *An*. *vagus* and *An*. *umbrosus* should be a point of concern as these species have similar breeding preferences such as rice paddies compared to *An*. *minimus* and *An*. *maculatus* [27] and thus may be exposed to similar insecticide pressure. The selection for insecticide resistance in the non-vectors mosquitoes raises concerns for the pyrethroid-based control in the future. Routine monitoring of insecticide resistance should be continued in Lao PDR as part of insecticide resistance management [28]. In addition we recommend the use of different insecticides with different modes of action and/or in rotation if IRS and LLINs are used in combination in the same area [29].

Our study showed that DDT resistance is widespread in *Anopheles* mosquitoes in Lao PDR hence confirming previous findings [8]. It has been suggested that DDT resistance may compromise vector control strategies using pyrethroid insecticides (i.e. ITNs and IRS) because of possible cross-resistance mechanism occurring between pyrethroids and DDT [4]. Cross resistance is generally conferred by *kdr* mutations that limits the fixation of DDT and pyrethroids to Sodium Gates Channel receptors [30,31]. The consequence is an augmentation of KDT_50_ coupled with low mortality rates in mosquitoes harboring *kdr* mutation after exposure to DDT and pyrethroids [32]. Here, due to a low number of mosquitoes tested and a low number of bioassays implemented per species and per insecticides, we did not find clear correlation between mortality rates and KDT_50_ for both DDT and pyrethroids in resistant species. It was then difficult to incriminate the role of *kdr* mutations in resistant mosquitoes. Sequencing of 279 mosquitoes surviving the exposure of insecticides confirmed this trend as no *kdr* alleles could be found at locus 1014 in all screened specimens hence suggesting an involvement of metabolic based resistance. Even if we cannot exclude the occurrence of mutations at other positions [33], increased metabolism due to overexpression of esterases, GSTs and P450 monooxygenases genes is likely. In DDT and pyrethroid resistant *Anopheles* species from Vietnam up-regulation of these 3 detoxification enzyme families were observed [18]. Rongnoparut et al. also reported increased mRNA expression of two P450 genes in a deltamethrin- resistant population of *An*. *minimus* in Thailand [34]. Research into insecticide resistance mechanisms should be encouraged to develop specific molecular tools for rapid detection of insecticide resistance markers in malaria vectors in the Mekong sub-region. This research will also help in decision making with regard to the use of insecticides to use in malaria control programmes.

The persistence of DDT resistance in *Anopheles* mosquitoes from Lao and neighboring countries raises some questions considering that DDT was banned > 25 years ago in the GMS [4,8]. In Lao PDR, DDT use was stopped in 1990 but officially banned in 2010 [5]. Although DDT has long residual life in the environment [35], we suspect that mosquito populations may still be exposed to DDT (or other xenobiotics having similar mode of action) hence exerting constant selection pressure on mosquitoes. The Ministry of Natural Resources and Environment Pollution Control Department reported recent illegal use of DDT in the Lao PDR [5]. A recent study carried out in Lao PDR and in other GMS countries showed the presence in various concentrations of organochlorine pesticides including DDT in sediment samples collected from the Lower Mekong River Basin and suggested recent use of DDT with regard to the ratio levels founds in the soils [36]. Tran and colleagues showed that DDT and its residues (DDD and DDE) were found in wetlands surrounding different types of land use such as rice field, fruit, eucalyptus and rubber plantations as well as uncultivated areas [36]. Agricultural pesticides (vegetables, rice fields, banana, etc.) and various xenobiotics (Agent Orange, illegal pesticides, fertilizers, etc.) are potential sources of contamination and resistance selection (Souris et al. submitted). In Thailand, the use of pesticides for crop protection showed to be significantly correlated with the presence of insecticide resistance in malaria vectors [37]. Clearly much work has to be done to understand the determinants associated with insecticide resistance in malaria vectors in the GMS.

## Conclusions

This 3 years entomology survey provided an important update of the resistance status of *Anopheles* species in Lao PDR which is in line with the Strategy for Malaria Elimination in the Greater Mekong Sub region by 2030 [38]. No pyrethroid resistance was found in primary malaria vectors indicating that the continuation of insecticide-treated bed net distribution and universal coverage should be encouraged in the country, especially in southern part of Lao PDR where malaria is endemic. However suspected resistance to permethrin was found in vector and high resistance in non-vector. Routine monitoring of the insecticide resistance levels and mechanisms should continue in Lao PDR to ensure effective malaria control in the country. Alterative tools have also to be deployed to better target early feeding and exophagic malaria vectors.

## Supporting information

S1 Table Insecticide susceptibility tests against *Anopheles* sp. from Lao PDR. (PDF)
