# Supplementary material for: Insecticide resistance status of malaria vectors in Lao PDR
Source: PLoS One. 2017 Apr 24;12(4):e0175984. doi: 10.1371/journal.pone.0175984 (PMC5402946; doi:10.1371/journal.pone.0175984)
Supplement: S1 Table — (PDF) [file pone.0175984.s001.pdf]

MALVEC project 2013-2015; Insecticide susceptibility tests, Anopheles sp., Lao PDR

Province	year	Season	Species	Insecticide	Lethal time 50	Lower limit	Upper limit	Lethal time 95	Lower limit	Upper limit	Slope	CI	p	n	n Control
Vientiane province	2014	dry	*An. minimus s.l.*	permethrin	13.2	13.0	13.4	21.2	20.5	22.1	8.0	+/- 0.3106	0.054	134	88
Vientiane province	2014	dry	*An. minimus s.l.*	deltamethrin	10.3	9.1	11.5	19.3	17.6	25.4	6.0	+/- 0.4542	0.015	173	88
Vientiane province	2014	dry	*An. minimus s.l.*	DDT	21.5	18.7	23.7	41.1	37.1	52.7	5.8	+/- 0.4673	0.041	97	88
Vientiane province	2014	dry	*An. aconitus*	permethrin	11.3	10.9	11.7	17.6	16.5	19.1	8.5	+/- 0.6103	0.547	98	62
Vientiane province	2014	dry	*An. aconitus*	deltamethrin	10.1	9.5	10.9	15.1	13.3	18.9	9.5	+/- 1.5837	0.200	91	62
Vientiane province	2014	dry	*An. aconitus*	DDT	27.1	25.3	28.9	43.4	39.0	51.6	8.0	+/- 1.0495	0.638	34	62
Saravane	2014	dry	*An. maculatus s.l.*	permethrin	13.7	12.7	14.6	24.7	22.0	29.6	6.4	+/- 0.8191	0.715	37	46
Saravane	2014	dry	*An. maculatus s.l.*	deltamethrin	9.3	8.6	10.2	21.0	17.9	26.6	4.7	+/- 0.5297	0.638	39	46
Saravane	2014	dry	*An. maculatus s.l.*	DDT	50.1	46.9	53.4	102.9	90.2	124.5	5.3	+/- 0.5423	0.984	30	46
Attapeu	2014	dry	*An. maculatus s.l.*	permethrin	11.7	10.3	13.3	25.5	22.8	36.5	4.9	+/- 0.4179	0.015	80	60
Attapeu	2014	dry	*An. maculatus s.l.*	deltamethrin	8.7	7.2	10.0	14.8	13.7	22.4	7.1	+/- 0.6385	0.018	78	60
Attapeu	2014	dry	*An. maculatus s.l.*	DDT	39.3	37.6	41.1	77.2	70.4	87.2	5.6	+/- 0.4103	0.253	77	60
Attapeu	2014	dry	*An. minimus s.l.*	permethrin	7.5	5.5	9.9	21.2	18.8	51.0	3.6	+/- 0.4219	0.008	41	37
Attapeu	2014	dry	*An. minimus s.l.*	deltamethrin	5.1	3.4	6.6	14.1	11.9	34.4	3.7	+/- 0.4591	0.040	40	37
Attapeu	2014	dry	*An. minimus s.l.*	DDT	n/a	n/a	n/a	n/a	n/a	n/a	n/a	n/a	n/a	n/a	37
Bokeo	2014	dry	*An. umbrosus*	permethrin	n/a	n/a	n/a	n/a	n/a	n/a	n/a	n/a	n/a	n/a	16
Bokeo	2014	dry	*An. umbrosus*	deltamethrin	9.8	8.8	10.9	24.3	20.0	33.2	6.8	+/- 1.0725	0.071	27	16
Bokeo	2014	dry	*An. umbrosus*	DDT	n/a	n/a	n/a	n/a	n/a	n/a	n/a	n/a	n/a	n/a	16
Phongsaly	2014	dry	*An. maculatus s.l.*	permethrin	14.0	13.1	15.0	25.4	22.5	30.5	6.4	+/- 0.7524	0.471	30	29
Phongsaly	2014	dry	*An. maculatus s.l.*	deltamethrin	12.9	11.7	14.0	22.5	19.5	28.9	6.8	+/- 1.0725	0.966	19	29
Phongsaly	2014	dry	*An. maculatus s.l.*	DDT	30.9	26.3	34.1	59.4	49.8	89.6	5.8	+/- 1.2678	0.982	20	29
Attapeu	2014	rainy	*An. vagus*	permethrin	13.7	12.6	14.7	30.1	26.3	36.7	4.8	+/- 0.4924	0.846	39	40
Attapeu	2014	rainy	*An. vagus*	deltamethrin	12.9	12.4	13.4	19.4	18.2	21.1	9.3	+/- 0.7734	0.124	56	40
Attapeu	2014	rainy	*An. vagus*	DDT	38.6	35.1	42.0	95.5	77.2	140.7	4.2	+/- 0.6527	0.983	36	40
Attapeu	2014	rainy	*An. nivipes s.l.*	permethrin	11.7	11.0	12.3	21.6	19.7	24.5	6.2	+/- 0.5738	0.454	64	26
Attapeu	2014	rainy	*An. nivipes s.l.*	deltamethrin	8.2	6.4	10.3	17.9	16.2	36.9	4.9	+/- 0.5606	0.020	45	26
Attapeu	2014	rainy	*An. nivipes s.l.*	DDT	26.4	22.6	28.6	42.3	37.5	56.8	8.0	+/- 1.8727	0.513	29	26
Sekong	2014	rainy	*An. vagus*	permethrin	14.8	13.9	15.6	20.9	19.2	24.3	10.9	+/- 1.6606	0.636	30	36
Sekong	2014	rainy	*An. vagus*	deltamethrin	12.2	11.6	12.9	23.6	21.4	26.8	5.8	+/- 0.4677	0.089	62	36
Sekong	2014	rainy	*An. vagus*	DDT	34.3	31.8	37.1	154.7	112.7	262.8	3.6	+/- 0.4645	0.713	70	36
Khammouane	2014	rainy	*An. philippinensis*	permethrin	10.6	9.5	11.6	35.2	28.5	47.9	3.1	+/- 0.3258	0.229	100	25
Khammouane	2014	rainy	*An. philippinensis*	deltamethrin	n/a	n/a	n/a	n/a	n/a	n/a	n/a	n/a	n/a	n/a	25
Khammouane	2014	rainy	*An. philippinensis*	DDT	64.7	56.1	80.7	346.5	218.2	752.6	2.3	+/- 0.2958	0.608	75	25
Khammouane	2014	rainy	*An. nivipes s.l.*	permethrin	n/a	n/a	n/a	n/a	n/a	n/a	n/a	n/a	n/a	n/a	50
Khammouane	2014	rainy	*An. nivipes s.l.*	deltamethrin	6.7	5.5	7.6	23.2	19.3	30.1	3.0	+/- 0.3378	0.201	75	50
Khammouane	2014	rainy	*An. nivipes s.l.*	DDT	n/a	n/a	n/a	n/a	n/a	n/a	n/a	n/a	n/a	25	50
Bokeo	2014	rainy	*An. kochi*	permethrin	22.3	15.2	27.0	67.8	50.4	143.8	3.4	+/- 0.8215	0.635	12	15
Bokeo	2014	rainy	*An. kochi*	deltamethrin	16.7	n/a	n/a	28.7	n/a	n/a	7.0	+/- 3.4901	0.218	10	15
Bokeo	2014	rainy	*An. kochi*	DDT	42.2	n/a	n/a	103.2	n/a	n/a	4.2	+/- 1.5881	0.503	10	15
Bokeo	2014	rainy	*An. vagus*	permethrin	21.4	12.8	26.8	71.2	49.7	222.0	3.2	+/- 0.8958	0.563	9	15
Bokeo	2014	rainy	*An. vagus*	deltamethrin	n/a	n/a	n/a	n/a	n/a	n/a	n/a	n/a	n/a	n/a	15
Bokeo	2014	rainy	*An. vagus*	DDT	49.0	n/a	n/a	150.1	n/a	n/a	3.4	+/- 4.0147	0.643	18	15
Bokeo	2014	rainy	*An. umbrosus*	permethrin	35.1	27.6	45.1	165.4	93.7	1000.0	2.4	+/- 0.6617	0.630	12	15
Bokeo	2014	rainy	*An. umbrosus*	deltamethrin	n/a	n/a	n/a	n/a	n/a	n/a	n/a	n/a	n/a	n/a	15

Bokeo	2014	rainy	*An. umbrosus*	DDT	74.2	n/a	n/a	244.0	n/a	n/a	3.2	+/-	1.8018	0.869	16	15
Phongsaly	2014	rainy	*An. maculatus s.l.*	permethrin	n/a	n/a	n/a	n/a	n/a	n/a	n/a		n/a	n/a	n/a	17
Phongsaly	2014	rainy	*An. maculatus s.l.*	deltamethrin	n/a	n/a	n/a	n/a	n/a	n/a	n/a		n/a	n/a	n/a	17
Phongsaly	2014	rainy	*An. maculatus s.l.*	DDT	35.9	32.2	40.0	72.0	59.2	105.2	5.4	+/-	0.9616	0.687	16	17
Phongsaly	2014	rainy	*An. minimus s.l.*	permethrin	14.2	12.2	16.6	31.8	24.1	64.5	4.7	+/-	1.0979	0.892	15	10
Phongsaly	2014	rainy	*An. minimus s.l.*	deltamethrin	n/a	n/a	n/a	n/a	n/a	n/a	n/a		n/a	n/a	n/a	10
Phongsaly	2014	rainy	*An. minimus s.l.*	DDT	n/a	n/a	n/a	n/a	n/a	n/a	n/a		n/a	n/a	n/a	10
Luang Prabang	2014	rainy	*An. hyrcanus*	permethrin	12.9	11.4	14.4	25.8	21.4	36.3	5.5	+/-	0.9080	0.806	15	29
Luang Prabang	2014	rainy	*An. hyrcanus*	deltamethrin	11.6	9.5	13.9	25.8	19.5	49.1	4.7	+/-	1.0446	0.668	13	29
Luang Prabang	2014	rainy	*An. hyrcanus*	DDT	34.5	n/a	n/a	79.8	n/a	n/a	4.5	+/-	1.9284	0.999	10	29
Bolikhamxay	2014	rainy	*An. nivipes s.l.*	permethrin	10.8	9.1	12.5	22.6	17.8	39.0	5.2	+/-	1.1331	0.732	11	15
Bolikhamxay	2014	rainy	*An. nivipes s.l.*	deltamethrin												15
Bolikhamxay	2014	rainy	*An. nivipes s.l.*	DDT	14.1	12.3	15.7	29.8	24.5	43.6	5.0	+/-	0.8966	0.579	16	15
Bolikhamxay	2014	rainy	*An. philippinensis*	permethrin	13.2	11.5	16.0	43.4	30.0	90.3	3.2	+/-	0.5595	0.258	24	16
Bolikhamxay	2014	rainy	*An. philippinensis*	deltamethrin												16
Bolikhamxay	2014	rainy	*An. philippinensis*	DDT	13.2	11.5	14.5	37.2	28.9	63.3	5.2	+/-	0.9818	0.683	29	16
Bolikhamxay	2014	rainy	*An. minimus s.l.*	permethrin	10.4	9.2	11.7	29.2	23.4	42.0	3.7	+/-	0.4889	0.818	25	16
Bolikhamxay	2014	rainy	*An. minimus s.l.*	deltamethrin	n/a	n/a	n/a	n/a	n/a	n/a	n/a		n/a	n/a	n/a	16
Bolikhamxay	2014	rainy	*An. minimus s.l.*	DDT	n/a	n/a	n/a	n/a	n/a	n/a	n/a		n/a	n/a	n/a	16
Vientiane province	2014	rainy	*An. hyrcanus*	permethrin	15.6	14.0	17.3	28.7	24.5	38.1	6.2	+/-	1.0162	0.555	29	30
Vientiane province	2014	rainy	*An. hyrcanus*	deltamethrin	5.5	4.1	6.8	36.8	24.4	77.7	2.0	+/-	0.3215	0.677	25	30
Vientiane province	2014	rainy	*An. hyrcanus*	DDT	36.6	28.4	48.5	73.7	78.5	179.6	5.4	+/-	0.6157	0.000	30	30
Vientiane province	2014	rainy	*An. nivipes s.l.*	permethrin	13.0	11.2	14.7	22.9	18.9	37.1	6.7	+/-	1.5750	0.373	25	30
Vientiane province	2014	rainy	*An. nivipes s.l.*	deltamethrin	3.8	n/a	n/a	8.1	n/a	n/a	5.1	+/-	0.9939	0.051	25	30
Vientiane province	2014	rainy	*An. nivipes s.l.*	DDT	19.7	15.4	23.9	38.4	33.2	61.0	5.7	+/-	0.5945	0.040	38	30
Vientiane province	2014	rainy	*An. aconitus*	permethrin	5.5	4.9	6.0	9.2	7.9	12.1	7.4	+/-	1.3174	0.129	31	62
Vientiane province	2014	rainy	*An. aconitus*	deltamethrin	3.7	3.1	4.3	8.0	6.3	13.1	4.9	+/-	0.9445	0.357	26	62
Vientiane province	2014	rainy	*An. aconitus*	DDT	24.4	21.1	26.7	45.7	39.0	64.0	6.0	+/-	1.1988	0.721	26	62
Vientiane province	2015	dry	*An. minimus s.l.*	permethrin	20.8	17.2	29.7	44.3	30.5	204.0	5.0	+/-	1.5989	0.750	12	9
Vientiane province	2015	dry	*An. minimus s.l.*	deltamethrin	n/a	n/a	n/a	n/a	n/a	n/a	n/a		n/a	n/a	n/a	9
Vientiane province	2015	dry	*An. minimus s.l.*	DDT	24.7	19.4	45.9	79.9	44.0	686.3	3.2	+/-	0.9505	0.103	17	9
Vientiane province	2015	dry	*An. aconitus*	permethrin	21.3	18.3	24.2	53.0	43.7	71.6	4.2	+/-	0.5579	0.149	17	9
Vientiane province	2015	dry	*An. aconitus*	deltamethrin	n/a	n/a	n/a	n/a	n/a	n/a	n/a		n/a	n/a	n/a	9
Vientiane province	2015	dry	*An. aconitus*	DDT	n/a	n/a	n/a	n/a	n/a	n/a	n/a		n/a	n/a	n/a	9
Bokeo	2015	dry	*An. minimus s.l.*	permethrin	n/a	n/a	n/a	n/a	n/a	n/a	n/a		n/a	n/a	n/a	15
Bokeo	2015	dry	*An. minimus s.l.*	deltamethrin	n/a	n/a	n/a	n/a	n/a	n/a	n/a		n/a	n/a	n/a	15
Bokeo	2015	dry	*An. minimus s.l.*	DDT	34.3	30.2	38.1	60.5	50.4	94.5	6.7	+/-	1.5489	0.832	14	15
Bokeo	2015	dry	*An. umbrosus*	permethrin	18.4	14.5	21.8	53.5	44.5	75.7	3.5	+/-	0.3548	0.035	116	15
Bokeo	2015	dry	*An. umbrosus*	deltamethrin	n/a	n/a	n/a	n/a	n/a	n/a	n/a		n/a	n/a	n/a	15
Bokeo	2015	dry	*An. umbrosus*	DDT	n/a	n/a	n/a	n/a	n/a	n/a	n/a		n/a	n/a	n/a	15
Khammouane	2015	dry	*An. vagus*	permethrin	19.5	17.6	21.1	31.2	27.6	39.2	8.0	+/-	1.3926	0.799	26	18
Khammouane	2015	dry	*An. vagus*	deltamethrin	n/a	n/a	n/a	n/a	n/a	n/a	n/a		n/a	n/a	n/a	18
Khammouane	2015	dry	*An. vagus*	DDT	43.5	38.6	48.7	91.2	71.2	175.2	5.1	+/-	1.2175	0.998	18	18
Phongsaly	2015	dry	*An. minimus s.l.*	permethrin	20.2	14.8	25.8	87.0	56.8	223.0	2.6	+/-	0.5418	0.188	10	13
Phongsaly	2015	dry	*An. minimus s.l.*	deltamethrin	n/a	n/a	n/a	n/a	n/a	n/a	n/a		n/a	n/a	n/a	13
Phongsaly	2015	dry	*An. minimus s.l.*	DDT	12.8	8.8	16.3	51.6	36.6	101.4	2.7	+/-	0.5215	0.989	12	13

Phongsaly	2015	dry	*An. maculatus s.l.*	permethrin	43.5	36.5	56.5	128.9	85.1	366.6	3.5	+/- 0.8000	0.306	17	15
Phongsaly	2015	dry	*An. maculatus s.l.*	deltamethrin	14.6	11.3	17.5	27.3	21.5	55.1	6.1	+/- 1.6992	0.469	10	15
Phongsaly	2015	dry	*An. maculatus s.l.*	DDT	23.4	20.5	26.9	46.5	37.2	74.2	5.5	+/- 1.0961	0.435	15	15
Luang Prabang	2015	dry	*An. maculatus s.l.*	permethrin	28.3	25.9	30.6	65.5	57.2	79.0	4.5	+/- 0.4326	0.552	35	39
Luang Prabang	2015	dry	*An. maculatus s.l.*	deltamethrin	12.4	9.9	15.4	30.8	22.2	69.9	4.1	+/- 0.9738	0.055	16	39
Luang Prabang	2015	dry	*An. maculatus s.l.*	DDT	45.5	39.2	57.6	137.1	113.5	303.9	3.4	+/- 0.4305	0.019	39	39
Luang Prabang	2015	dry	*An. minimus s.l.*	permethrin	14.0	11.2	16.8	32.4	24.5	60.6	4.5	+/- 0.9823	0.168	13	41
Luang Prabang	2015	dry	*An. minimus s.l.*	deltamethrin	n/a	n/a	n/a	n/a	n/a	n/a	n/a	n/a	n/a	n/a	41
Luang Prabang	2015	dry	*An. minimus s.l.*	DDT	31.2	26.8	37.5	73.1	54.2	144.6	4.5	+/- 0.9419	0.288	12	41
Luang Prabang	2015	dry	*An. vagus*	permethrin	16.1	14.6	17.6	35.4	31.7	40.8	4.8	+/- 0.4224	0.270	47	49
Luang Prabang	2015	dry	*An. vagus*	deltamethrin	8.4	6.6	9.9	20.9	17.1	29.1	4.1	+/- 0.6451	0.263	21	49
Luang Prabang	2015	dry	*An. vagus*	DDT	104.2	75.7	368.0	339.8	159.4	7470.5	3.2	+/- 0.9949	0.939	46	49
Phongsaly	2015	rainy	*An. maculatus s.l.*	permethrin	22.0	17.9	26.4	100.8	69.8	196.1	2.5	+/- 0.3914	0.168	19	15
Phongsaly	2015	rainy	*An. maculatus s.l.*	deltamethrin	n/a	n/a	n/a	n/a	n/a	n/a	n/a	n/a	n/a	n/a	15
Phongsaly	2015	rainy	*An. maculatus s.l.*	DDT	46.0	33.9	79.6	688.7	245.9	10975.6	1.4	+/- 0.3320	0.985	17	15
Phongsaly	2015	rainy	*An. kochi*	permethrin	26.1	21.6	30.3	65.1	51.6	99.3	4.1	+/- 0.7032	0.690	11	15
Phongsaly	2015	rainy	*An. kochi*	deltamethrin	n/a	n/a	n/a	n/a	n/a	n/a	n/a	n/a	n/a	n/a	15
Phongsaly	2015	rainy	*An. kochi*	DDT	0 after 60 min	0 after 60 mir	0 after 60 min	0 after 60 min	0 after 60 mir	0 after 60 mir	0 after 60 r	0 after 60 mi	0 after 6	0 after	15
Bokeo	2015	rainy	*An. vagus*	permethrin	15.7	14.2	17.0	33.9	31.2	38.1	4.9	+/- 0.2226	0.003	144	70
Bokeo	2015	rainy	*An. vagus*	deltamethrin	24.7	23.5	25.8	65.6	60.6	72.0	3.9	+/- 0.1845	0.415	136	70
Bokeo	2015	rainy	*An. vagus*	DDT	56.9	54.4	60.2	118.4	104.6	139.4	5.2	+/- 0.3660	0.942	148	70
Vientiane province	2015	rainy	*An. nivipes s.l.*	permethrin	23.3	20.1	26.2	56.0	47.3	72.3	4.3	+/- 0.5445	0.288	17	15
Vientiane province	2015	rainy	*An. nivipes s.l.*	deltamethrin	15.2	n/a	n/a	38.5	n/a	n/a	4.1	+/- 1.5323	0.043	16	15
Vientiane province	2015	rainy	*An. nivipes s.l.*	DDT	48.9	44.6	57.1	90.2	70.9	168.3	6.2	+/- 1.4385	0.053	20	15
Vientiane province	2015	rainy	*An. aconitus*	permethrin	16.2	13.7	17.8	23.0	20.4	32.4	10.7	+/- 2.9434	0.104	16	15
Vientiane province	2015	rainy	*An. aconitus*	deltamethrin	n/a	n/a	n/a	n/a	n/a	n/a	n/a	n/a	n/a	n/a	15
Vientiane province	2015	rainy	*An. aconitus*	DDT	22.3	18.9	25.5	69.2	55.8	95.9	3.3	+/- 0.4134	0.486	19	15
Vientiane province	2015	rainy	*An. tessellatus*	permethrin	22.3	18.0	27.8	50.4	36.6	127.0	4.6	+/- 1.2330	0.653	9	15
Vientiane province	2015	rainy	*An. tessellatus*	deltamethrin	n/a	n/a	n/a	n/a	n/a	n/a	n/a	n/a	n/a	n/a	15
Vientiane province	2015	rainy	*An. tessellatus*	DDT	n/a	n/a	n/a	n/a	n/a	n/a	n/a	n/a	n/a	n/a	15
Attapeu	2015	rainy	*An. nivipes s.l.*	permethrin	14.7	12.0	17.4	45.9	35.7	70.2	3.3	+/- 0.5038	0.874	20	20
Attapeu	2015	rainy	*An. nivipes s.l.*	deltamethrin	22.3	19.1	25.2	61.6	51.2	81.2	3.7	+/- 0.4566	0.723	21	20
Attapeu	2015	rainy	*An. nivipes s.l.*	DDT	87.6	n/a	n/a	210.4	n/a	n/a	4.3	+/- 2.6144	0.854	20	20
Attapeu	2015	rainy	*An. vagus*	permethrin	15.7	12.8	18.4	50.1	40.5	68.9	3.3	+/- 0.4114	0.979	19	14
Attapeu	2015	rainy	*An. vagus*	deltamethrin	n/a	n/a	n/a	n/a	n/a	n/a	n/a	n/a	n/a	n/a	14
Attapeu	2015	rainy	*An. vagus*	DDT	2070.5	n/a	n/a	4475638.2	n/a	n/a	0.5	+/- 0.3733	1.000	15	14
Attapeu	2015	rainy	*An. kochi*	permethrin	25.1	21.8	28.0	52.8	44.9	69.1	5.1	+/- 0.7585	0.962	17	15
Attapeu	2015	rainy	*An. kochi*	deltamethrin	n/a	n/a	n/a	n/a	n/a	n/a	n/a	n/a	n/a	n/a	15
Attapeu	2015	rainy	*An. kochi*	DDT	76.8	n/a	n/a	173.7	n/a	n/a	4.6	+/- 2.2149	0.970	14	15
Sekong	2015	rainy	*An. kochi*	permethrin	n/a	n/a	n/a	n/a	n/a	n/a	n/a	n/a	n/a	n/a	25
Sekong	2015	rainy	*An. kochi*	deltamethrin	n/a	n/a	n/a	n/a	n/a	n/a	n/a	n/a	n/a	n/a	25
Sekong	2015	rainy	*An. kochi*	DDT	27.7	24.8	30.6	65.4	55.2	84.1	4.4	+/- 0.5120	0.525	23	25
Sekong	2015	rainy	*An. nivipes s.l.*	permethrin	18.4	16.7	20.1	41.5	37.1	48.0	4.7	+/- 0.4036	0.744	50	50
Sekong	2015	rainy	*An. nivipes s.l.*	deltamethrin	12.1	10.5	13.5	27.3	24.1	32.3	4.7	+/- 0.5319	0.723	49	50
Sekong	2015	rainy	*An. nivipes s.l.*	DDT	36.0	34.4	37.8	77.0	69.9	87.1	5.0	+/- 0.3375	0.533	77	50
Luang Prabang	2015	rainy	*An. nivipes s.l.*	permethrin	19.6	17.7	21.4	54.5	48.1	63.8	3.7	+/- 0.2834	0.329	46	50

Luang Prabang	2015	rainy	*An. nivipes s.l.*	deltamethrin	19.5	n/a	n/a	106.3	n/a	n/a	2.2	+/-	0.8536	0.504	28	50
Luang Prabang	2015	rainy	*An. nivipes s.l.*	DDT	23.4	17.6	28.4	68.8	57.4	111.2	3.5	+/-	0.3541	0.019	44	50
Luang Prabang	2015	rainy	*An. minimus s.l.*	permethrin	11.4	9.7	13.0	33.6	28.3	42.8	3.5	+/-	0.3737	0.503	37	50
Luang Prabang	2015	rainy	*An. minimus s.l.*	deltamethrin	n/a	n/a	n/a	n/a	n/a	n/a	n/a	n/a		n/a	n/a	50
Luang Prabang	2015	rainy	*An. minimus s.l.*	DDT	26.6	24.8	28.2	47.5	43.3	53.8	6.5	+/-	0.6089	0.236	41	50
